# Marked changes in diversity and relative activity of picoeukaryotes with depth in the world ocean

**DOI:** 10.1038/s41396-019-0506-9

**Published:** 2019-10-23

**Authors:** Caterina R. Giner, Massimo C. Pernice, Vanessa Balagué, Carlos M. Duarte, Josep M. Gasol, Ramiro Logares, Ramon Massana

**Affiliations:** 1 Institut de Ciències del Mar (CSIC), Passeig Marítim de la Barceloneta, 37-49, ES-08003 Barcelona, Catalonia Spain; 2 Institute for the Oceans and Fisheries, University of British Columbia, AERL, 2202 Main Mall, Vancouver, BC, V6T 1Z4, Canada; 3 Red Sea Research Center (RSRC) and Computational Biosciences Research Center (CBRC), King Abdullah University of Science and Technology (KAUST), Thuwal, 23955-6900 Saudi Arabia; 4 Centre for Marine Ecosystem Research, School of Science, Edith Cowan University, Joondalup, WA Australia

**Keywords:** Microbial ecology, Molecular ecology, Community ecology

## Abstract

Microbial eukaryotes are key components of the ocean plankton. Yet, our understanding of their community composition and activity in different water layers of the ocean is limited, particularly for picoeukaryotes (0.2–3 µm cell size). Here, we examined the picoeukaryotic communities inhabiting different vertical zones of the tropical and subtropical global ocean: surface, deep chlorophyll maximum, mesopelagic (including the deep scattering layer and oxygen minimum zones), and bathypelagic. Communities were analysed by high-tthroughput sequencing of the 18S rRNA gene (V4 region) as represented by DNA (community structure) and RNA (metabolism), followed by delineation of Operational Taxonomic Units (OTUs) at 99% similarity. We found a stratification of the picoeukaryotic communities along the water column, with assemblages corresponding to the sunlit and dark ocean. Specific taxonomic groups either increased (e.g., Chrysophyceae or Bicosoecida) or decreased (e.g., Dinoflagellata or MAST-3) in abundance with depth. We used the rRNA:rDNA ratio of each OTU as a proxy of metabolic activity. The highest relative activity was found in the mesopelagic layer for most taxonomic groups, and the lowest in the bathypelagic. Altogether, we characterize the change in community structure and metabolic activity of picoeukaryotes with depth in the global ocean, suggesting a hotspot of activity in the mesopelagic.

## Introduction

Protists are key components of marine microbial communities, playing a central role in marine food webs [1], particularly in carbon cycling [2]. Despite their importance, the distribution and activity of protists in the global ocean is still poorly understood. In particular, little is known about protists communities in the dark ocean, as most efforts have focused in protists populating the photic layer. Yet, the deep ocean (>1000 m depth) is a huge biome, being the largest reservoir of organic carbon in the ocean [3], and containing about 70% of the ocean’s prokaryotic cells [2].

The water column is divided into the epipelagic (0–200 m depth), mesopelagic (200–1000 m), and bathypelagic (1000–4000 m) layers [2]. The sunlit epipelagic zone harbors photosynthetic microbes and is the scenario of the classic oceanic food web, whereas the dark ocean (i.e., mesopelagic and bathypelagic regions) is characterized by no light, high pressure, low temperature, and high inorganic nutrient content [2]. A global survey indicated that the distribution of protists in the upper ocean is predominantly structured by oceanographic basin, pointing to dispersal limitation [4, 5], while in the bathypelagic layer, protist assemblages appear to be structured by water masses [6]. Previous studies on the distribution of protists along the water column are limited to a few specific oceanic regions and indicate a clear differentiation between epipelagic and deep ocean communities [7–14].

The mesopelagic layer represents ~20% of the global ocean and is an area of intense remineralization [2], believed to sustain a large fish biomass [15]. It includes a layer few hundred meters thick known as the ‘deep scattering layer’ (DSL), characterized by the accumulation of large stocks of mesopelagic fish and migrant zooplankton [15], leading to intense biological activity. Furthermore, in regions like the eastern tropical Pacific Ocean and the tropical Indian Ocean, the mesopelagic zone contains layers with very low oxygen concentration, known as oxygen minimum zones (OMZs), which play a key role in the nitrogen cycle and contain specific microbial communities [16]. However, protist communities in OMZ or anoxic basins have received less attention than those in oxygenated mesopelagic waters [17–20] and changes in protist relative activity in the DSL or OMZ at the global scale remain unknown.

DNA-based analyses have contributed greatly to elucidate global protist community structure in the sunlit [4] and dark ocean [6]. Yet, sequences obtained using DNA include metabolically inactive or dead cells, while sequences obtained from RNA extracts derive from the actual ribosomes and therefore from living cells. In addition, as the ribosomal content is regulated to match the protein synthesis needs during population growth and acclimation [21], the rRNA:rDNA ratio for a given taxon has been used as a proxy for their relative activity. This approach has been applied to microbial eukaryotes in a few epipelagic [7, 22, 23] and vertical profile [8, 12] regional studies, but it has not yet been applied at the global scale.

Here, we present the first global survey investigating vertical changes in picoeukaryote community composition and relative activity using Illumina sequencing of 18S rRNA genes amplified from DNA and RNA extracts. We analyzed samples from 7 depths (from surface to 4000 m) in 13 stations encompassing the Atlantic, Indian and Pacific Oceans sampled during the Malaspina-2010 Circumnavigation expedition [24], targeting protists <3 µm in size (picoeukaryotes). Specifically, we analysed changes in picoeukaryotic community structure and relative activity along the ocean water column, assessing environmental factors driving these changes. Furthermore, we tested whether picoeukaryote community structure and relative activity differ in the OMZ and DSL zones.

## Materials and methods

### Sample collection and nucleic acid extraction

During the Malaspina-2010 Circumnavigation expedition (December 2010–July 2011), a total of 91 water samples were collected in 13 stations distributed across the world’s oceans (Fig. S1, Table S1). Each station was sampled at seven different depths with Niskin bottles attached to a CTD profiler that had sensors for conductivity, temperature, salinity, and oxygen. Each vertical profile included samples at surface (3 m), deep chlorophyll maximum (DCM), and 2–3 depths in mesopelagic (200–1000 m) and bathypelagic (1000–4000 m) waters. Samples for inorganic nutrients (NO_3_– NO_2_–, PO_4_^3−^, and SiO_2_) were collected from the Niskin bottles, kept frozen, and measured spectrophotometrically using an Alliance Evolution II autoanalyzer [25]. Along the cruise, different deep-water masses were sampled. The proportion of the different deep-water masses in each sample was inferred from its temperature, salinity, and oxygen concentration [26].

For each sample, about 12 liters of seawater was prefiltered through a 200 µm nylon mesh to remove large plankton and then sequentially filtered using a peristaltic pump through a 20 µm nylon mesh and then through 3 and 0.2 µm pore-size polycarbonate filters of 142 mm diameter (Isopore, Millipore). Filtration time was about 15–20 min. The filters were flash frozen in liquid nitrogen and stored at −80 °C until DNA and RNA extraction. Polycarbonate filters containing the 0.2–3 µm size fraction were cut into small pieces and cryogrinded with a Freezer-Mill 6770 (Spex) using three cycles of 1 min. Then, RNA and DNA were extracted simultaneously using the Nucleospin RNA kit (Macherey-Nagel) plus the NucleoSpin RNA/DNA Buffer Set (Macherey-Nagel) procedures. The presence of residual DNA in RNA extracts was checked by PCR with universal eukaryotic primers and, if detected, was removed using the Turbo DNA-free kit (Applied Biosystems). RNA was reverse transcribed to cDNA using the SuperScript III reverse Transcriptase (Invitrogen) and random hexamers. DNA and cDNA extracts were quantified with a Qubit 1.0 (Thermo Fisher Scientific) and kept at −80 °C.

### Sequencing and processing of picoeukaryotic community

Picoeukaryotic diversity was assessed by amplicon sequencing of the V4 region of the 18 S rDNA gene (~380 bp) using the Illumina MiSeq platform and paired-end reads (2 × 250 bp). PCR amplifications with the eukaryotic universal primers TAReukFWD1 and TAReukREV3 [27] and amplicon sequencing were carried out at the Research and Testing Laboratory (Lubbock, USA; http://www.researchandtesting.com). Illumina reads obtained from DNA and cDNA extracts (rDNA and rRNA sets, respectively) were processed together following an in-house pipeline [28] at the Marine Bioinformatics Service (MARBITS) of the Institut de Ciències del Mar. Briefly, raw reads were corrected using BayesHammer [29] as indicated by Schirmer et al. [30]. Corrected paired-end reads were subsequently merged with PEAR [31] and sequences longer than 200 bp were quality-checked and dereplicated using USEARCH [32]. OTU (Operational Taxonomic Unit) clustering at 99% similarity was done using UPARSE v8 [33]. Chimera check and removal was performed both *de novo* and using the SILVA reference database [34]. Taxonomic assignment was done by BLAST searches against PR2 [35] and two in-house marine protist databases (available at https://github.com/ramalok) based on a collection of Sanger sequences from environmental surveys [36] and of 454 reads from the BioMarKs project [23]. Metazoan, Charophyta, and nucleomorphs OTUs were removed. The final OTU table contained 79 rDNA samples and 90 rRNA samples (some samples were excluded due to suboptimal PCR or sequencing) accounting for 11,570,044 reads clustered into 45,115 OTUs. To enable comparisons between samples, the OTU table was randomly subsampled down to the minimum number of reads per sample (22,379 reads) using the *rrarefy* function in the *Vegan* package [37]. This turned into a final table including 38,300 OTUs and 3,782,051 reads. Sequences are available at the European Nucleotide Archive with Accession number PRJEB23771 (http://www.ebi.ac.uk/ena).

### Statistical analyses

Statistical analyses were performed in R (R Core Team [38]) using the package *Vegan* [37]. Bray–Curtis dissimilarities were used as an estimator of beta diversity, which were then clustered using non-metric multidimensional scaling (NMDS). In NMDS, the differences between predefined groups were statistically tested with ANOSIM using 1,000 permutations. PERMANOVA analyses were performed to determine the proportion of the variation in community composition that was explained by the measured environmental variables. The Shannon index (H') and richness (number of OTUs) were calculated as estimators of alpha diversity.

To assess the relative activity of taxonomic groups, rRNA:rDNA ratios were calculated for each OTU in each sample by dividing the relative abundance of the OTU in the RNA between their relative abundance in the DNA. OTUs occurring in only one of the two datasets were not considered. Each individual ratio provided an indication of the relative activity of a given OTU in a given sample. Ratios from the same taxonomic group and water layer were analyzed together. Specifically, ratios >1 indicated metabolically “*hyperactive*” taxa, ratios <1 indicated metabolically “*hypoactive*” taxa, while ratios ~1 pointed to taxa with “*average*” activity levels. Differences in rRNA:rDNA ratios by water layer were evaluated using a Wilcoxon test.

OTUs present in the four layers and showing preferential activity in one layer (200 OTUs) were aligned with MAFFT [39]. A phylogenetic tree was inferred with RAxML [40] using the generalized time reversible (GTR) model of nucleotide substitution considering a CAT/Gamma-distributed rate of variation across sites (including 20 rate categories). The best topology out of 1,000 pseudoreplicates was kept. The amount of branch length associated to the different water layers as estimated by gUniFrac [41], was used as an estimate of the amount of phylogenetic diversity associated to them. gUniFrac was run with an alpha value of 0.5.

Some mesopelagic samples were taken in the OMZ or in the DSL. For specific analyses, DSL samples ( 9 samples) were compared with the remaining mesopelagic samples (22 samples). DSL was identified by acoustic data [15], and DSL samples were selected within the mesopelagic samples according to the scattering profile. OMZ samples ( 8 samples) were considered as those where oxygen concentration was <2 mg O_2_ L^−1^ [42], and were also compared with the remaining oxygenated mesopelagic samples (23 samples).

## Results

### Community structure along the water column

**Fig. 1:**
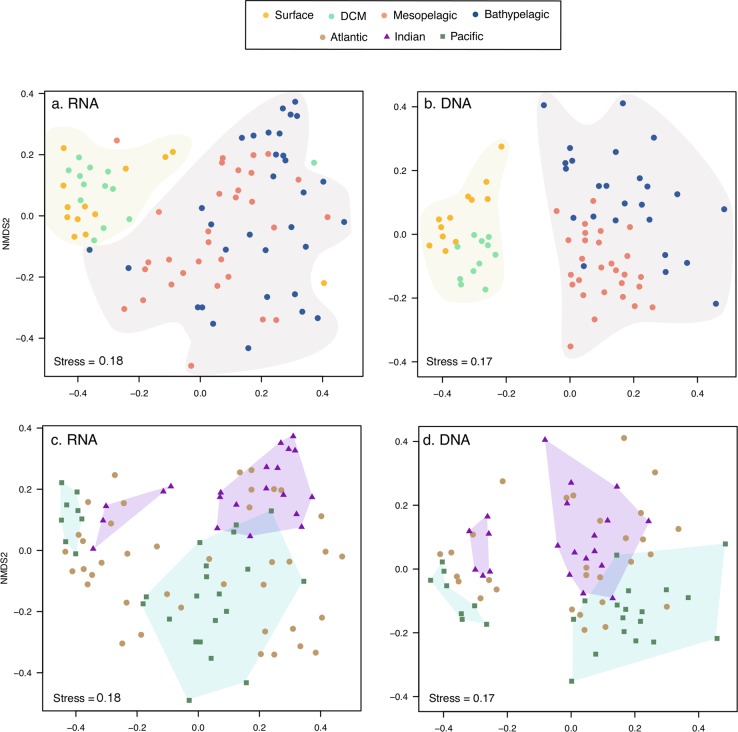
Comparison of picoeukaryotic community structure derived from the rRNA (**a**, **c**) and rDNA (**b**, **d**) datasets based on a non-metric multidimensional scaling analysis (NMDS). Each sample is colored according to the depth layer (**a**, **b**) and has a different symbol shape according to the ocean where it comes from **(c**, **d**). Samples from  the photic and aphotic layers are shadowed (a, b). Samples from the Indian and the Pacific oceans in the photic and aphotic layers were grouped in separate polygons (c, d).

The diversity of picoeukaryotic assemblages decreased significantly with depth in both rRNA and rDNA datasets (Wilcoxon test *p* < 0.05, Fig. S2). This trend was also observed when analyzing the Atlantic, Indian, and Pacific oceans separately (Fig. S3), but higher diversity was found in the Pacific rRNA mesopelagic layer compared with that in other oceans. Comparing community structure of all samples in NMDS revealed different clusters for rDNA and rRNA, also separating photic and aphotic communities within each one (Fig. S4). Specific NMDS for rDNA and rRNA datasets supported a significant differentiation between photic and aphotic communities (ANOSIM: *R*^2^ = 0.52, *p* < 0.001 for rRNA and *R*^2^ = 0.72, *p* < 0.001 for rDNA, Fig. 1a, b). Within the photic zone, surface and DCM communities formed two groups (ANOSIM: *R*^2^ = 0.39 for rRNA and *R*^2^ = 0.60 for rDNA, *p* < 0.001), while the mesopelagic and bathypelagic communities did not constitute different groups (ANOSIM: *R*^2^ = 0.14 and *R*^2^ = 0.35, *p* < 0.004, Fig. 1). Picoeukaryotic communities from the Indian and Pacific oceans formed differentiated clusters within each water layer, while Atlantic communities were intermixed among the Indian and Pacific communities (Fig. 1c, d, Fig. S5).

Environmental parameters changed markedly along the water column (Fig. S6, Table S1), likely exerting environmental selection. The variance in community structure explained by the measured environmental variables was analyzed with PERMANOVA. Light (taken as presence or absence) was the most important environmental factor structuring picoeukaryotic community, accounting for about 15% of community variance in both rRNA and rDNA datasets, while temperature, oxygen, the ocean basin (Atlantic, Indian, or Pacific), and depth explained each 3–7% of community variance along the water column. About 55–60% of the variance remained unexplained by the measured parameters (Table S2). Individual PERMANOVA tests for the photic and aphotic zone indicated that environmental factors explained 65% of the variance in community structure in the photic zone, whereas water mass explained about 25% of community structure variance in the dark ocean (Table S2).

### Horizontal community structure

**Fig. 2:**
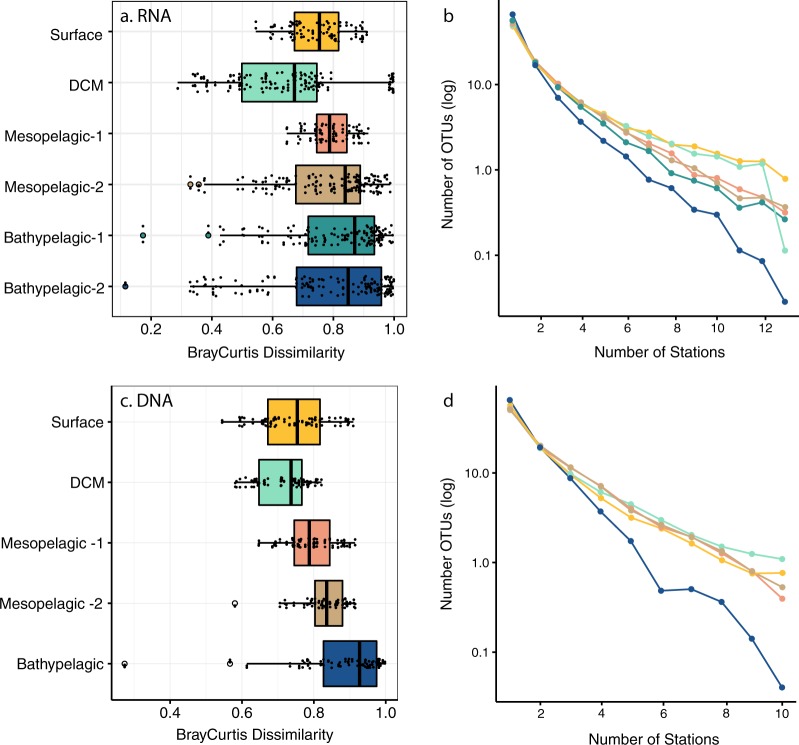
Community similarity and prevalence of OTUs across ocean depth layers, based on rRNA (upper panels) and rDNA (lower panels). **a**, **c** Distribution of Bray–Curtis dissimilarities values among samples from a given layer. **b**, **d** Number of OTUs within each water layer detected in a varying number of stations. Boxplot colors indicate the different water layers. The color lines (b, d) correspond to the boxplot colors

We analysed the change in community composition within specific depth layers, which provides an indication of the connectivity between communities. We compared one sample of each water layer (i.e., six layers in rRNA and five in rDNA, as mesopelagic and bathypelagic samples were divided into two sublayers) among all stations (13 stations in rRNA and  10 in rDNA). Communities within the photic zone were more similar among stations (Bray–Curtis distance 0.7, Fig. 2a, c) than those in the dark ocean, with those in the deepest bathypelagic layer being the most dissimilar among stations (median Bray–Curtis values ~0.9). Interestingly, community composition in the bathypelagic was highly heterogeneous, as shown by the wide range of Bray–Curtis dissimilarities (ranging from 0.1 to 1.0). In addition, most of the OTUs within each layer were found in a single station (Fig. 2b, d), with a decline in the number of OTUs with increasing prevalence (number of stations where a given OTU was present). This OTU decline was not equal for all layers, being steepest in the bathypelagic. For instance, 3.3% of surface-OTUs were present in at least 80% of the samples, but this was ten times lower (0.23% of OTUs) for the bathylepagic zone. This pattern of decreasing prevalence with depth is consistent with the higher Bray–Curtis dissimilarity of bathypelagic communities. The most prevalent OTUs were also the most abundant ones (data not shown).

We also evaluated the potential vertical dispersal of OTUs in the water column. A total of 13% of OTUs (considering only those represented by >5 reads) in the rRNA dataset were restricted to the mesopelagic layer, compared with 4–6% of unique OTUs in the remaining depth layers (Table S3). A similar pattern was observed in the rDNA dataset, including a larger fraction of unique OTUs in the mesopelagic. Most shared OTUs were shared within the dark ocean (i.e., mesopelagic and bathypelagic layers) or the photic zone (i.e., surface and DCM).

### Taxonomic change with depth

In general, there was a good correlation in the relative abundances of picoeukaryotic groups observed in the rDNA and rRNA datasets (Fig. S7). Exceptions were MALV-I and MALV-II (Marine Alveolate groups I and II) and Polycystinea, highly overrepresented in the rDNA (altogether 64.9% of rDNA reads but only 6.9% of rRNA reads). Mamiellophyceae and Basidiomycota were also overrepresented in the rDNA dataset. Ciliophora was highly overrepresented in the rRNA survey (11.6% of rRNA reads versus 0.2% of rDNA reads), together with MOCH-5 (Marine Ochrophyta group 5), Ancyromonadida and MAST-12 (Marine Stramenopile group 12, Fig. S7).

**Fig. 3:**
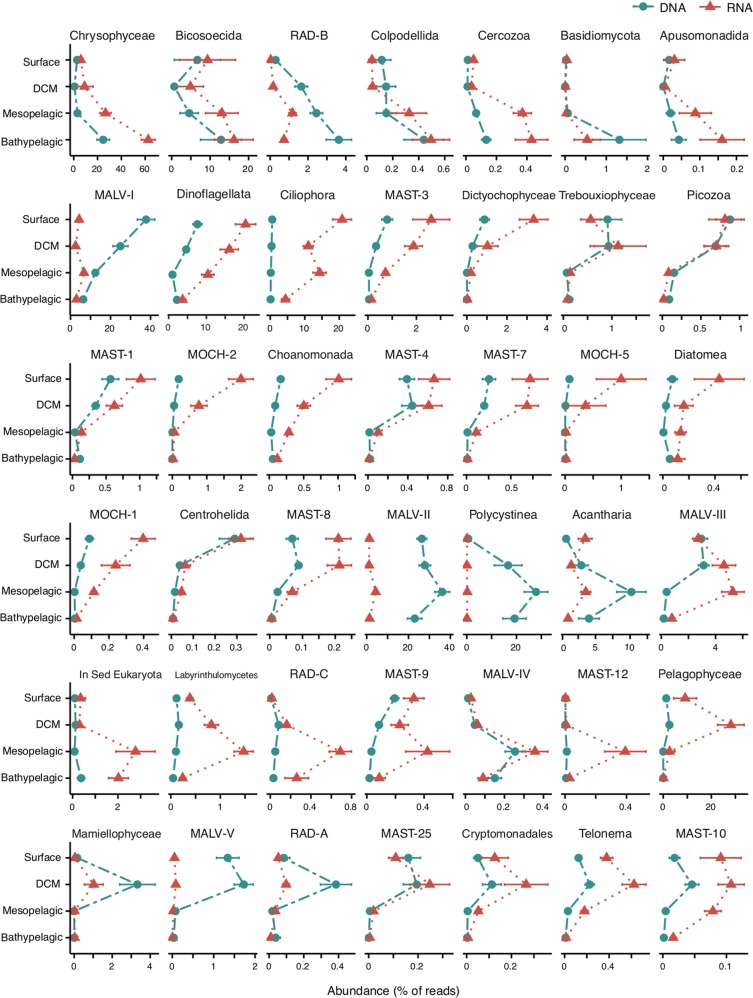
Averaged relative abundance of the main taxonomic groups in the four water layers derived from rRNA (red line) and rDNA (blue line) surveys. Groups are ordered first based on the layer of their maximal abundance, and then by their read abundance

We observed four patterns of taxonomic change with depth: (i) groups that increased their abundance with depth (Chrysophyceae, Bicosoecida, and RAD-B [Radiolaria group B]), (ii) groups that decreased their abundance with depth (Dinoflagellata, Ciliophora, and all MAST and MOCH clades), (iii) groups that peaked in the mesopelagic (Labyrinthulomycetes, RAD-C, and MALV-IV), and (iv) groups that peaked at the DCM (Pelagophyceae, Mamiellophyceae, Telonema, and Cryptomonadales) (Fig. 3). This resulted in-group depth-dependent differences, with Ciliophora and Dinoflagellata dominating in surface waters (42% of reads), Pelagophyceae and Dinoflagellata at the DCM (44% of reads), and Chrysophyceae and Bicosoecida in the dark ocean (40% of reads in the mesopelagic and 73% in the bathypelagic).

Among the different ocean basins all groups followed the same pattern of vertical changes (Fig. S8), except two groups: MALV-III peaks in the mesopelagic in the Pacific Ocean, whereas in the other two oceans it decreases with depth, and RAD-B shows opposite trends between Atlantic and Indian oceans (increasing and decreasing their abundance with depth, respectively). Chrysophyceae was the most abundant group in all deep basins, representing 28% of reads in Atlantic, 56% of the reads in the Indian and 19% of the reads in the Pacific oceans. The second most abundant taxonomic group was Bicosoecida for the Indian and Atlantic Ocean and Ciliophora in the Pacific.

### Changes in the relative activity with depth

**Fig. 4:**
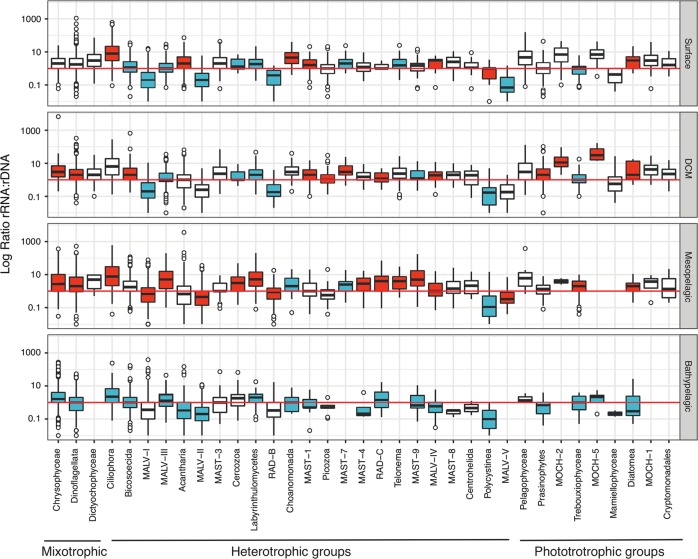
Distribution of the activity ratios (rRNA:rDNA) for all OTUs within major taxonomic groups in the four investigated depth layers. The red line indicates a ratio of 1. For each group, the layers with significantly higher or lower values between them, according to Wilcoxon tests, are colored in red or blue, respectively. Groups with no boxplot color (e.g., MAST-3), indicate no significant differences among layers. Note that the taxonomic groups are listed according to their main trophic mode (mixotrophic, heterotrophic or phototrophic)

To determine changes in the relative activity, we calculated the ratio of rRNA vs. rDNA reads for all OTUs (31,866 ratios). The photic (surface, DCM) and mesopelagic layers had a median rRNA:rDNA ~1 for all OTUs, pointing to no deviations in relative activity (Fig. S9). In contrast the median rRNA:rDNA was <1 in the bathypelagic zone (Fig. S9), pointing to a lower fraction of metabolically active cells in deeper waters when compared with overlying waters. This lower relative activity in the bathypelagic was observed in the majority of the taxonomic groups (Fig. 4), which in general displayed the lowest activity in the bathypelagic (except MALV-I, Cercozoa, Labyrinthulomycetes, RAD-B, and Telonema that showed the lowest activity at the photic zone). Many groups displayed higher relative activity in the mesopelagic (MALV-I, MALV-III, MALV-II, Cercozoa, Labyrinthulomycetes, among others, Fig. 4). Most phototrophic groups showed the higher relative activity at the DCM layer, except for Trebouxiophyceae that displayed higher relative activity in the mesopelagic (Fig. 4). No major differences were found in relative activity among ocean basins, although activities tended to be higher in the Pacific Ocean than in the Indian Ocean for the aphotic layers (Fig. S10). Overall, relative activities were more variable with depth than among ocean basins.

**Fig. 5:**
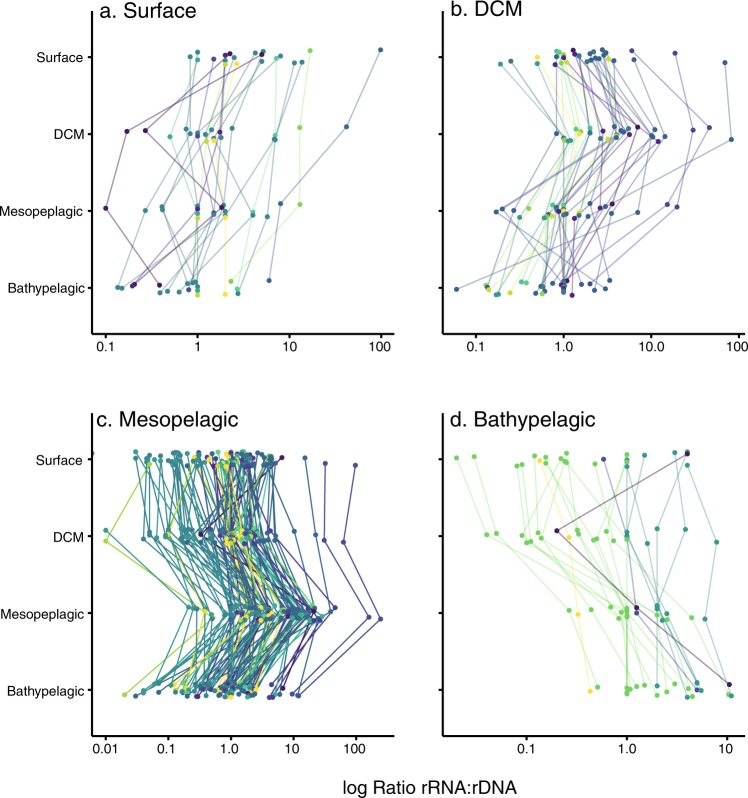
Activity of the OTUs present in the 4 water layers classified according to the layer where they present the highest activity. **a** OTUs more active in surface, **b** at DCM, **c** at the mesopelagic, and **d** at the bathypelagic. Colors indicate different taxonomic groups

Further exploration was done in 200 OTUs present at the 4 depths, which represented 2.2% of the analyzed OTUs and 48.8% of the reads. Out of these 200 OTUs, 121 showed their highest activity in the mesopelagic (Fig. 5) being most of them classified as Dinoflagellata and MALV-I (25 and 20% of the OTUs active in the mesopelagic, respectively). We individually analyzed the OTUs of the four most abundant groups, Chrysophyceae, MALV-I, Dinoflagellata, and Bicosoecida (Fig. S11). Interestingly whereas most of the OTUs of Chrysophyceae and MALV-I showed their highest activity in the mesopelagic, there was a high variability in Dinoflagellata and Bicosoecida OTUs. The phylogenetic tree of the 200 OTUs showed that the different clades did not contain exclusively OTUs active in a particular depth, OTUs from the same taxonomic group but having their maximum of activity in different depths were closely related in the tree. In addition, we analysed the phylogenetic diversity between the different water layers. OTUs from surface and DCM were the most phylogenetically similar (UniFrac distance 0.5), whereas bathypelagic OTUs were the most phylogenetically different (UniFrac 0.7, Fig. S12).

### Patterns in OMZ and DSL

We sampled the OMZ and DSL in specific stations (eight samples of three Pacific Ocean stations were sampled in the OMZ, and DSL was sampled in nine of the 13 stations). Whereas no clear differentiation was observed between DSL and the non-DSL mesopelagic communities (Fig. S13a), OMZ communities were more similar among them and differed significantly from the oxygenated mesopelagic samples (Fig. S13b). No differences in richness were found between the DSL and the non-DSL communities, while the OMZ had a higher richness than the oxygenated mesopelagic samples (Fig. S14).

**Fig. 6:**
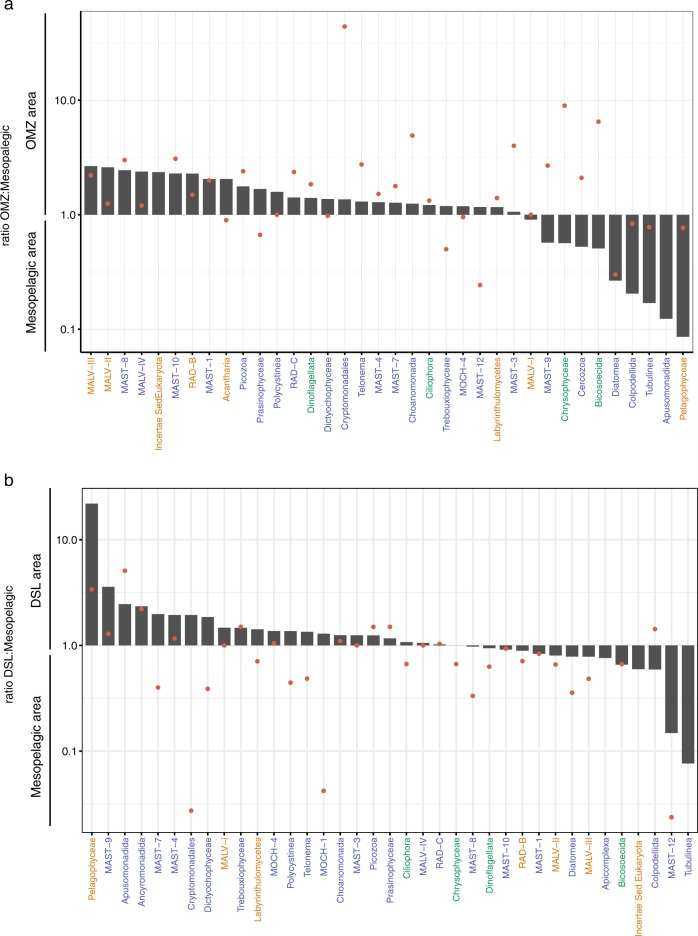
Comparison of relative abundance of taxonomic groups and their relative activity in the communities sampled within the Oxygen Minimum Zone (**a**) and DSL (**b**) with respect to the rest of mesopelagic samples. Bars represent the ratio between the abundance of specific groups among the two regions, while dots represent the ratio of the relative activity (rRNA:rDNA). Groups are ordered by their higher abundance ratio in the OMZ region (**a**) and DSL region (**b**) (only groups with an abundance >0.05% in the total mesopelagic realm are shown). Note that bars or dots in the OMZ zone of the plot mean higher abundance or activity in that zone. Group colors represent the relative abundance of the group in the total mesopelagic: abundance >10% in green, 1–10% in orange, and 0.5–1% in blue

We explored which taxonomic groups preferred the OMZ as compared with the fully oxygenated mesopelagic waters (Fig. 6a). Groups accounting for the 47% of the total mesopelagic reads showed a preference for the OMZ. This groups include: Ciliophora, Dinoflagellata, MALV-III, MALV-II, and Acantharia. Groups enriched in the OMZ tended to have higher activities (rRNA:rDNA ratio) in that region, except for Chrysophyceae and Bicosoecida, which were more active but less abundant in the OMZ (Fig. 6a). Two OTUs of Crysophyceae with contrasting behavior explained this difference in the Chrysophyceae, one being very abundant in the OMZ and the other in the oxygenated mesopelagic, whereas no clear pattern was found for Bicosoecida. The group formed by unclassified OTUs (*IncertaeSedis* Eukaryota), which could potentially include new species within high-rank taxa, showed a higher abundance and relative activity in the OMZ, pointing to potential taxonomic novelty. A similar analysis of DSL samples yielded inconclusive results (Fig. 6b). Some taxonomic groups were enriched in the DSL (MALV-I, Pelagophyeae, MAST-9), but the relative abundance ratio was, in general, smaller for most groups, indicating no clear DSL preference. Surprisingly, most groups showed lower activities at the DSL, suggesting that the DSL is not a specific and selective habitat for picoeukaryotes, in contrast with the OMZ that influences picoeukaryotic community structure and induces changes in their relative activity.

## Discussion

Our work provides the first global assessment of the change in community structure and relative activity of picoeukaryotes from surface down to 4000 m. The Malaspina expedition was taking samples during 7 months and the cruise was organized so that most regions were sampled at similar meteorological seasons. We found clear patterns of diversity and relative activity change along the water column, with clear differences in picoeukaryotic assemblages at the community level between the photic and aphotic regions across the different oceans, which is consistent with the results obtained from previous regional surveys [11, 14]. Overall, epipelagic communities were more similar among themselves than communities in the dark ocean, suggesting a higher dispersal in surface and DCM layers, likely due to faster currents in the upper ocean (Villarino et al. [43]) when compared with the deep ocean. Bathypelagic assemblages, which were the most different across the ocean (Fig. 2), seemed to be structured by water masses, which explained 25% of the variability in their community structure. Thus, two distinct water masses, even geographically close, may contain different communities and viceversa [6]. Furthermore, more OTUs were shared between the two deep-ocean layers than between the two epipelagic.

The abundance of the different taxonomic groups changed with depth, with Chrysophyceae, Bicosoecida, Radiolaria, and Colpodellida increasing in relative abundance with depth, as reported in earlier regional studies done in the North Pacific and North Atlantic ocean [7, 10, 11, 14, 44]. RAD-C (Radiolaria group C) was an important component of twilight and dark ocean communities, showing a peak in relative abundance in the mesopelagic layer. Radiolaria are typically very large in terms of cell size, so, they were not expected to be relevant in our picoplankton dataset. Yet, Radiolaria can be fragile, so they can break during size fractionation, and can also produce swarmers of picoplankton size, so their detection in picoplanktonic studies is still controversial. On the other hand, most photosynthetic groups (e.g., Pelagophyceae, and green algae) declined in abundance with depth, similarly to several heterotrophic lineages such as MAST clades or Picozoa. The occasional detection of metabolically active phototrophic groups in the deep ocean (e.g., Diatoms), which have already been detected sometimes at significant abundances in the dark ocean [12, 45], could be due to their attachment to rapidly sinking particles (e.g., aggregates,  faecal pellets [45], although some of the detected taxa may be mixotrophs (e.g., Dinoflagellata, Chrysophyceae), as this lifestyle is more common in the global ocean than currently acknowledged [46, 47].

Even though a moderate correlation of rDNA and rRNA relative abundances was found for most taxonomic groups, some groups were overrepresented in the rRNA dataset, whereas others like MALV-I, MALV-II, Polycystinea, and Acantharia were overrepresented in the rDNA dataset. It is known that MALV-I and MALV-II are usually dominant groups in rDNA surveys [4, 6, 9, 23, 48], whereas they may be up to ten times less abundant in rRNA surveys [23]. They likely have many rDNA operon copies [49] indicating that, in most cases, rDNA-based assessments may overestimate their abundance. The large variation in the rDNA copy number in eukaryotes, roughly related to cell size [50] and genome size [51], plus the reads that might be derived from dissolved extracellular DNA could affect the relative abundance observed in rDNA surveys, whereas rRNA is assumed to be absent from the extracellular pool [21]. In a previous study, we showed that sequencing surveys based on DNA and RNA extracts provide reasonable views of relative abundance, with different results depending on the taxonomic group [52]. However, since the rDNA operon copy number is an intrinsic feature of each taxa, we assumed that it was maintained within the whole taxonomic group and decided to focus on the changes of the rRNA:rDNA ratios of each group among the four water layers, aiming to identify a peak of activity for each group. This is an innovative aspect of the research presented here since only few studies have also included rRNA extracts [7, 12, 22, 23, 53]. Interestingly, many heterotrophic groups showed their highest relative activity in the mesopelagic layer across the three ocean basins, where oxyclines may promote hotspots of metabolic activity [54], and large carbon transfer by vertical migratory fish and invertebrates may promote relatively high microbial prey abundance to be grazed by metabolically active communities of Ciliates, Dinoflagellates, and Cercozoans, which were some of the most active groups in the mesopelagic layer (Fig. 6). Furthermore, it has been observed that the clearance rates of heterotrophic nanoflagellates were higher in mesopelagic than in epipelagic samples [55]. Relative activities of Cercozoa, MALV-I, and MALV-II were also high in the mesopelagic, in agreement with observations by Xu et al. [12]. Also, Hu et al. [7] found higher metabolic activity of Ciliophora at those depths. On the other hand, as expected, the majority of taxa were less active in the bathypelagic. Interestingly, some phototrophic groups showed activity in aphotic depths, indicating that they may be ingesting prey (i.e., mixotrophy). It has been shown that increases in the relative activity of some photosynthetic groups (e.g., pelagophytes, dinoflagellates) in the absence of light may indicate grazing activity (mixotrophy); some mixotrophic algae conduct phagotrophy when the availability of light or inorganic nutrients is reduced (Stoecker et al. [47], 56). Our analysis found depth-related patterns in the relative activity of most of the picoeukaryote groups and shed light on poorly known groups, such as MALV-III, highlighting the potential importance of protists in the deep ocean.

The mesopelagic layer contained the highest number of unique picoeukaryotic OTUs, in agreement with previous regional observations [11], but despite this, the mesopelagic was not the most diverse layer, as richness was higher in surface waters and decreased with depth, also coincident with previous studies [11, 14]. The larger number of OTUs exclusively present in the mesopelagic could be partially attributable to the existence of waters with specific conditions, such as the OMZ. Oxygen concentration has a strong influence on microbial distributions, and can affect the community structure of protists with only a subset of taxa able to adapt to anoxic or microoxic conditions [17–19, 57, 58]. Our results indicated that the richness found in OMZ in the Pacific Ocean is similar to that of epipelagic waters, as observed by Jing et al. [17], indicating a relatively high diversity in hypoxic waters. The most abundant groups in the OMZ retrieved with the rRNA were Chrysophyceae, Ciliates and Dinoflagellates. The presence of Chrysophyceae in the OMZ has been reported before [17], together with that of anaerobic Ciliates (e.g., mesodiniids) and Dinoflagellates [18, 19, 58]. The OMZ contains a diverse community of picoeukaryotes, with 25 different taxonomic groups present, most of them showing higher relative abundances (Fig. 6). Furthermore, the fact that most of the taxonomic groups were metabolically active could be explained by the high bacterivory activity of mixotrophic and heterotrophic groups, as bacterial abundance is typically high below the oxycline ([17], Fenchel and Finlay [59]), where ciliates are recognized as important grazers [19]. Besides, species richness was higher in the OMZ than in the mesopelagic zone, a pattern previously observed by Parris et al. [19] but that differs from other reports [18]. Yet, contrary to what we expected, we did not find conspicuous picoeukaryotic assemblages in the DSL as compared with the rest of mesopelagic samples, and intriguing observation that deserves further explorations.

In conclusion, this study provides the first insights on the changes in diversity and relative activity of picoeukaryotes along the whole water column at a global scale. Picoeukaryotic community structure was strongly differentiated in the water column, with two main communities corresponding to the epipelagic and the dark ocean. Our analysis identified the mesopelagic layer as an activity hotspot for picoeukaryotes, indicating also differentiated communities within OMZs.

## Supplementary information

Supplementary material
