# Supplementary material for: Marked changes in diversity and relative activity of picoeukaryotes with depth in the world ocean
Source: ISME J. 2019 Oct 23;14(2):437–49. doi: 10.1038/s41396-019-0506-9 (PMC6976695; doi:10.1038/s41396-019-0506-9)
Supplement: Supplementary file 1 — Supplementary material [file 41396_2019_506_MOESM1_ESM.pdf]

SUPPLEMENTARY FIGURES

Figure S1. World map showing the location of the Malaspina stations sampled for this study. Red triangles represent sations containing Oxygen Minimum Zone (OMZ) samples.

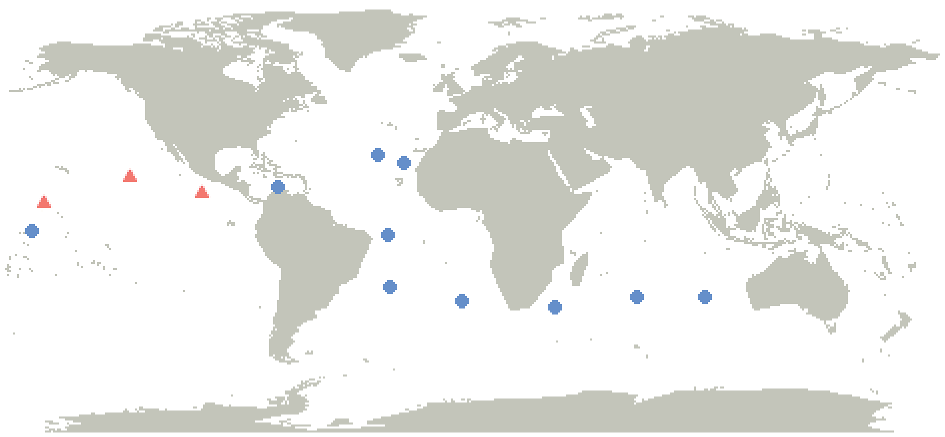

Figure S2. Alpha diversity of picoeukaryotic communities in the different water layers using the rRNA (upper boxplots) and rDNA (lower boxplots) datasets. (a) OTU richness and (b) Shannon Index (H'). Significant differences were found between photic and aphotic layers..

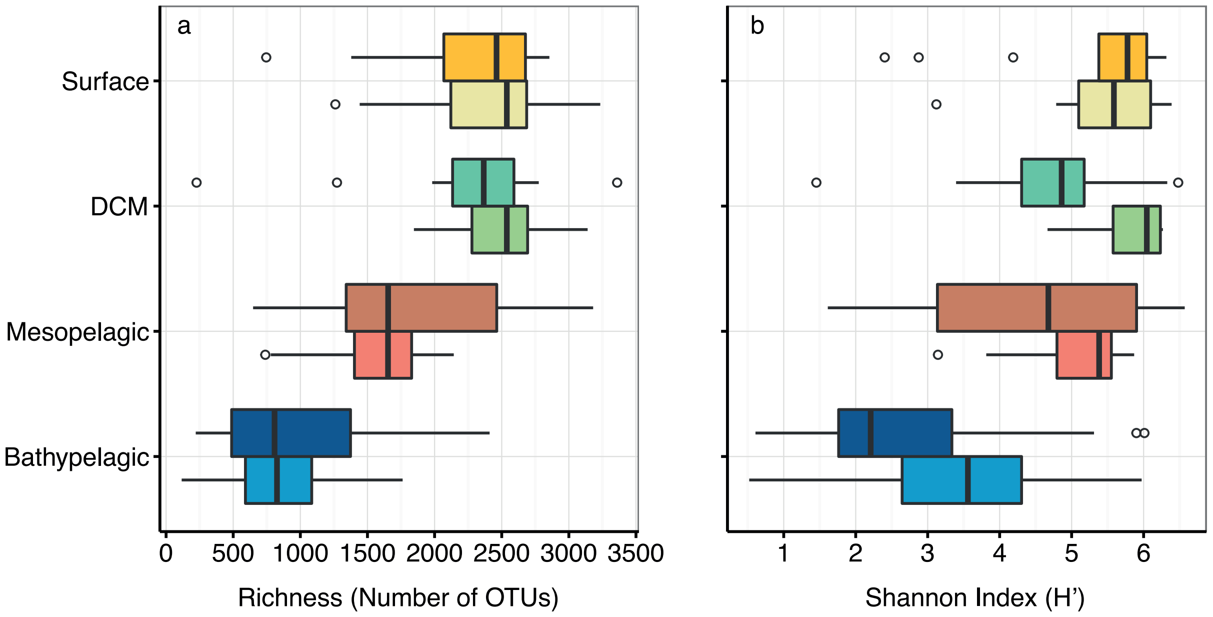

Figure S3. Richness of picoeukaryotic communities in samples from different water layers of each ocean basin using the rRNA (upper boxplots) and rDNA (lower boxplots) datasets.

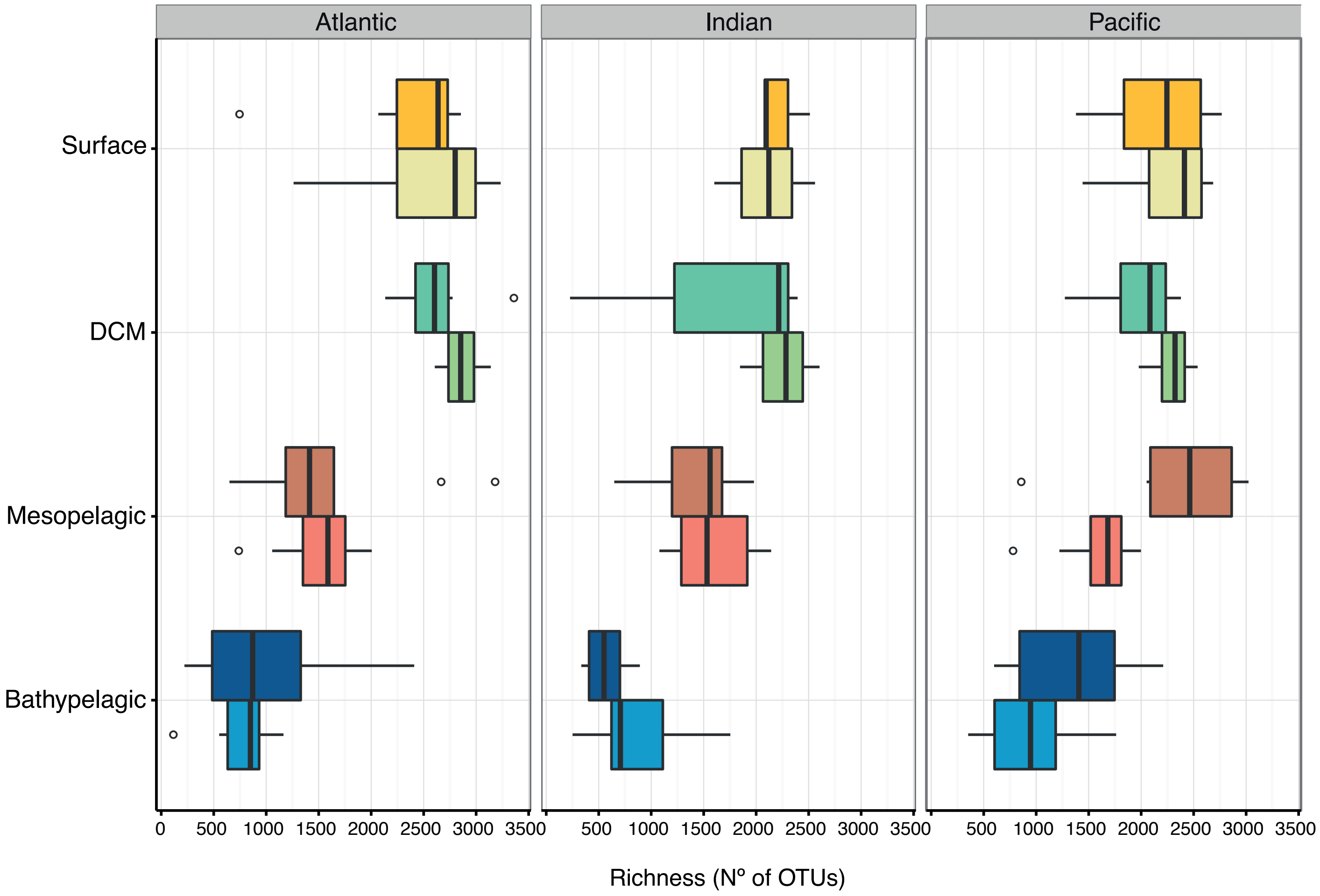

Figure S4. Clustering of all picoeukaryotic samples in a non-metric multidimensional scaling (NMDS) plot. Each sample is colored according to the depth layer and has different symbol shape according to the type of dataset (rDNA or rRNA).

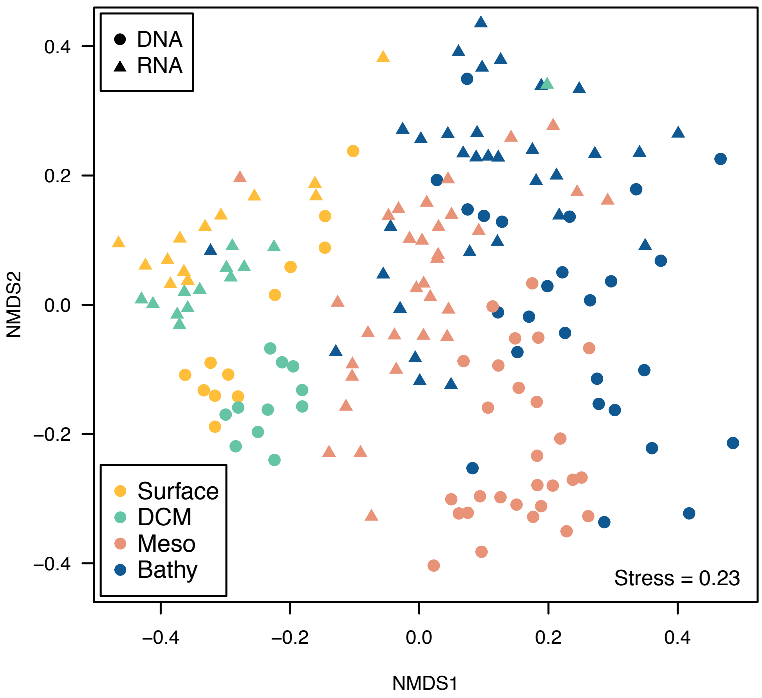

Figure S5. Dendograma of the picoeukaryotic samples for the RNA and DNA dataset. Each sample is colored according to their ocean.

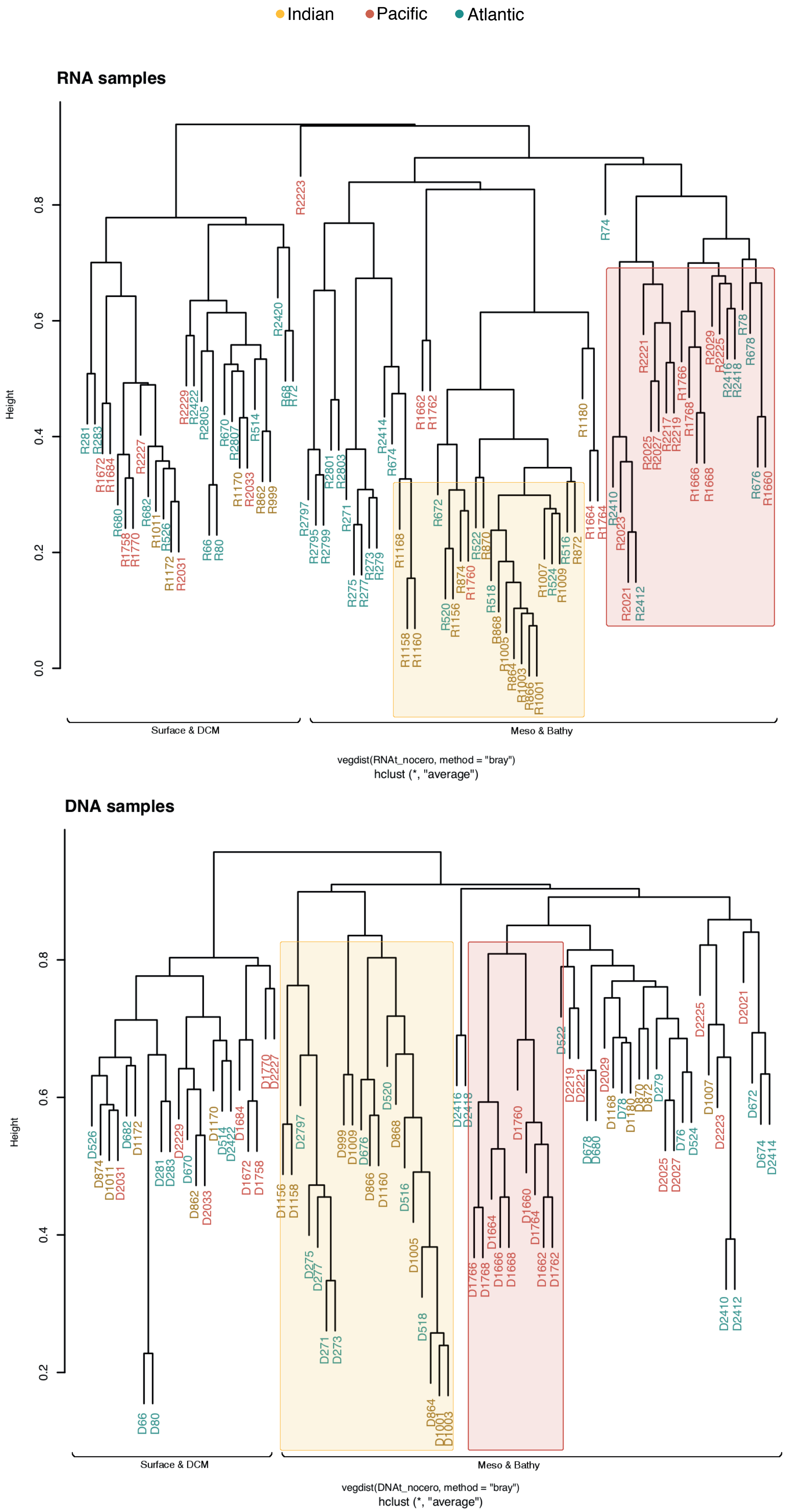

Figure S6. Main environmental variables and inorganic nutrient concentrations in the four layers of the water column (actual values as black dots and averaged values as brown lines).

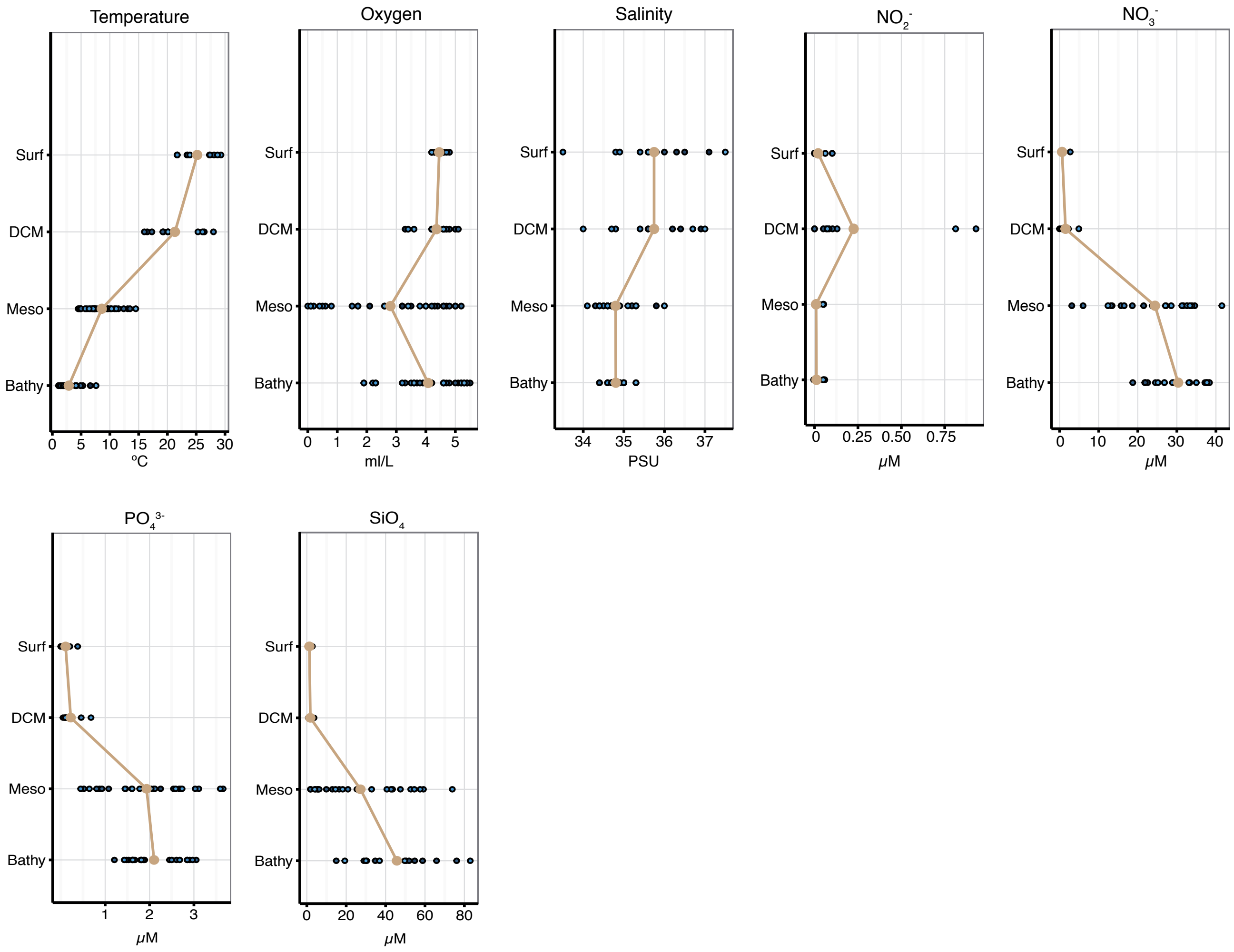

Figure S7. Representation of all phylogenetic groups based in the total abundance in the rRNA and rDNA surveys.

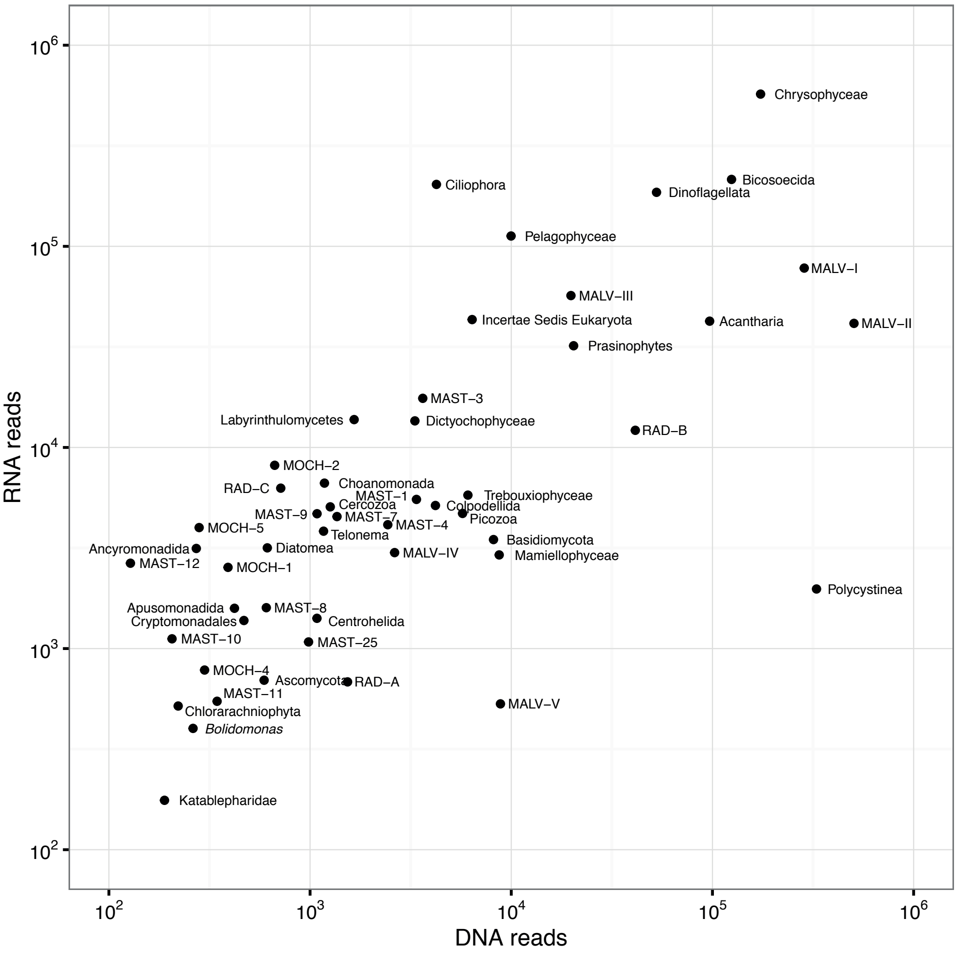

Figure S8. Relative abundance of the main taxonomic groups in the four water layers in the different ocean basins.

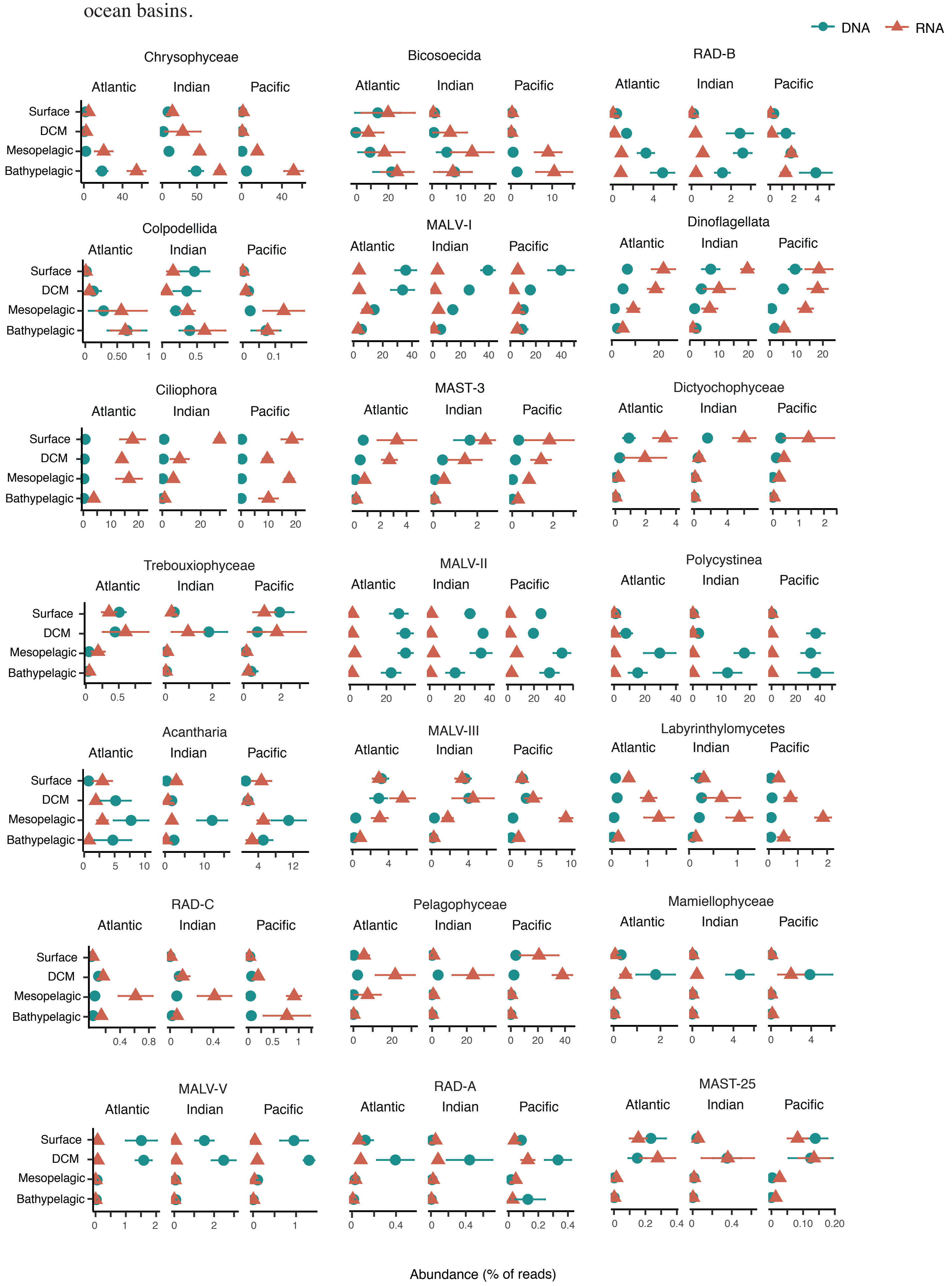

Figure S9. Distribution of the rRNA:rDNA ratios for all OTUs within a given depth layer. The red line indicates a ratio of 1.

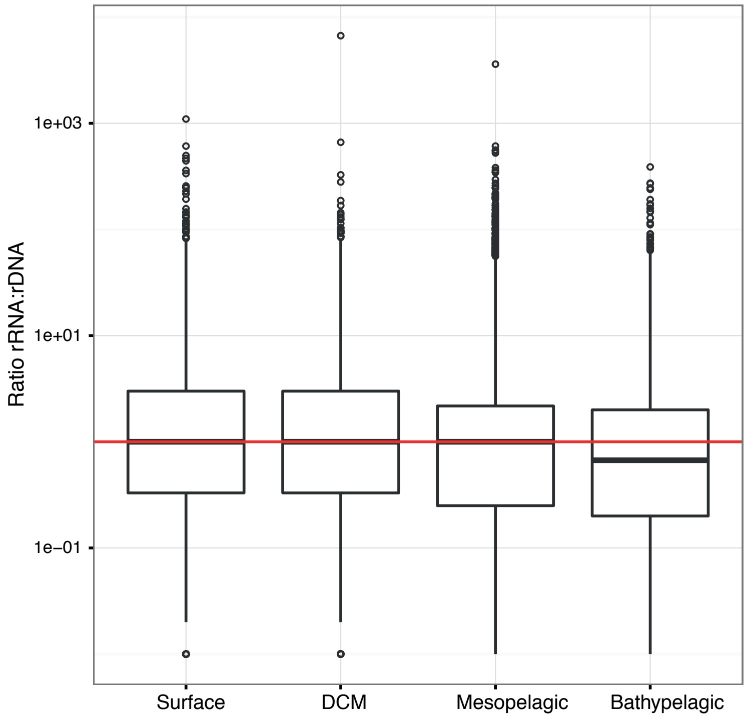

Figure S10. Distribution of the activity ratios (rRNA:rDNA) for all OTUs within major taxonomic groups in the four depth layers and the three oceans. The red line indicates a ratio of 1.

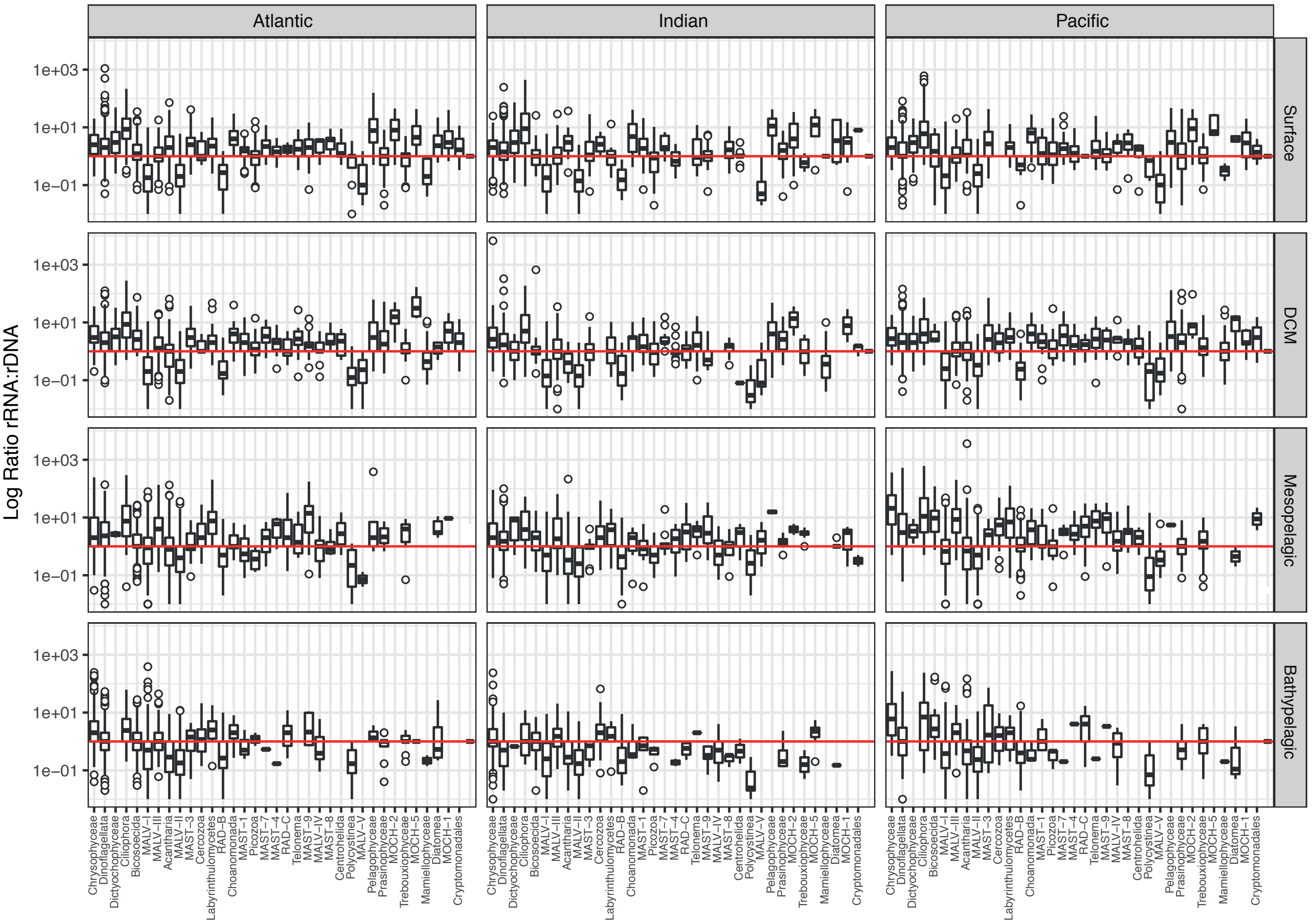

Figure S11. Activity ratios (rRNA:rDNA) of the OTUs of the four main taxonomic groups present at the four depths (surface, DCM, mesopelagic and bathypelagic).

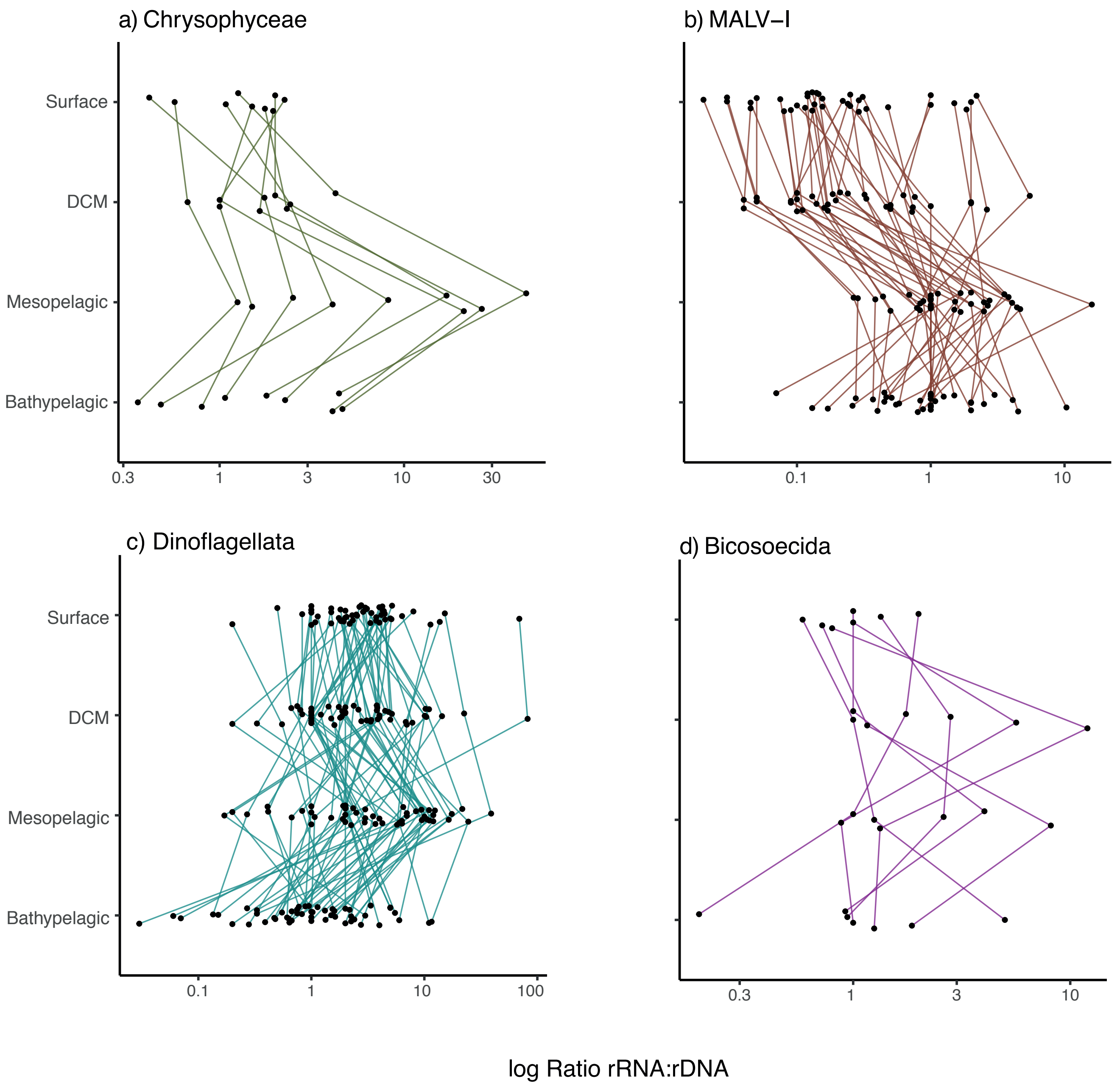

Figure S12. UPGMA dendogram based on the gUniFrac distances between samples from different water layers.

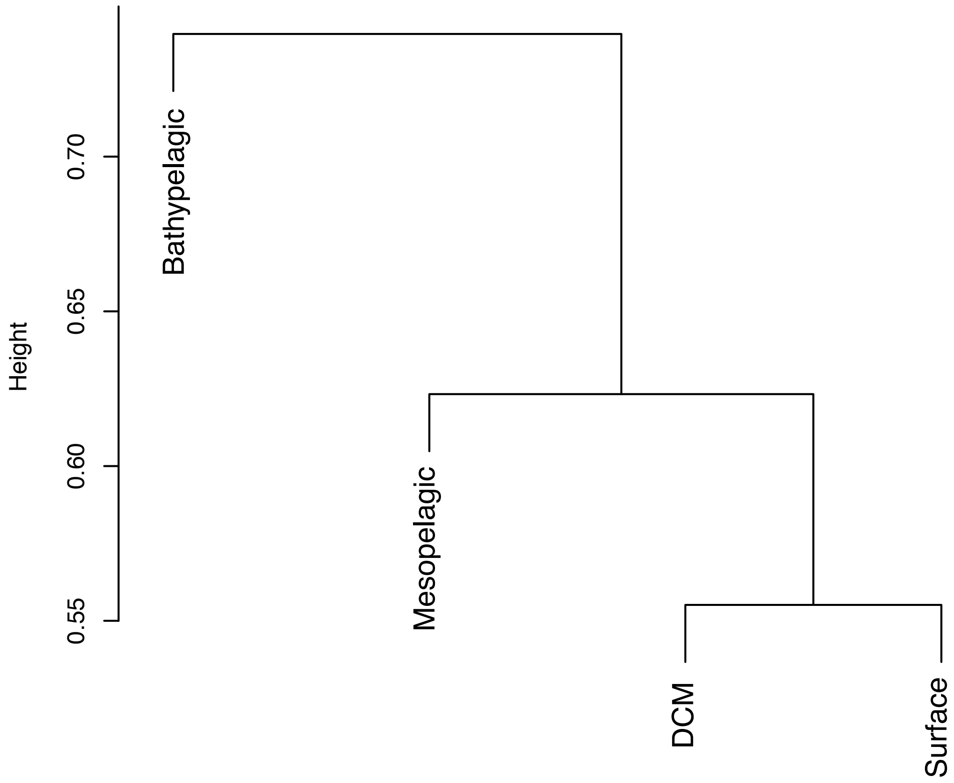

Figure S13. Clustering of all picoeukaryotic samples on a Non-metric multidimensional analysis (NMDS) based on Bray-Curtis dissimilarities differentiating between DSL (a, c) and OMZ (b, d) samples. Each sample is colored according the specific depth layer.

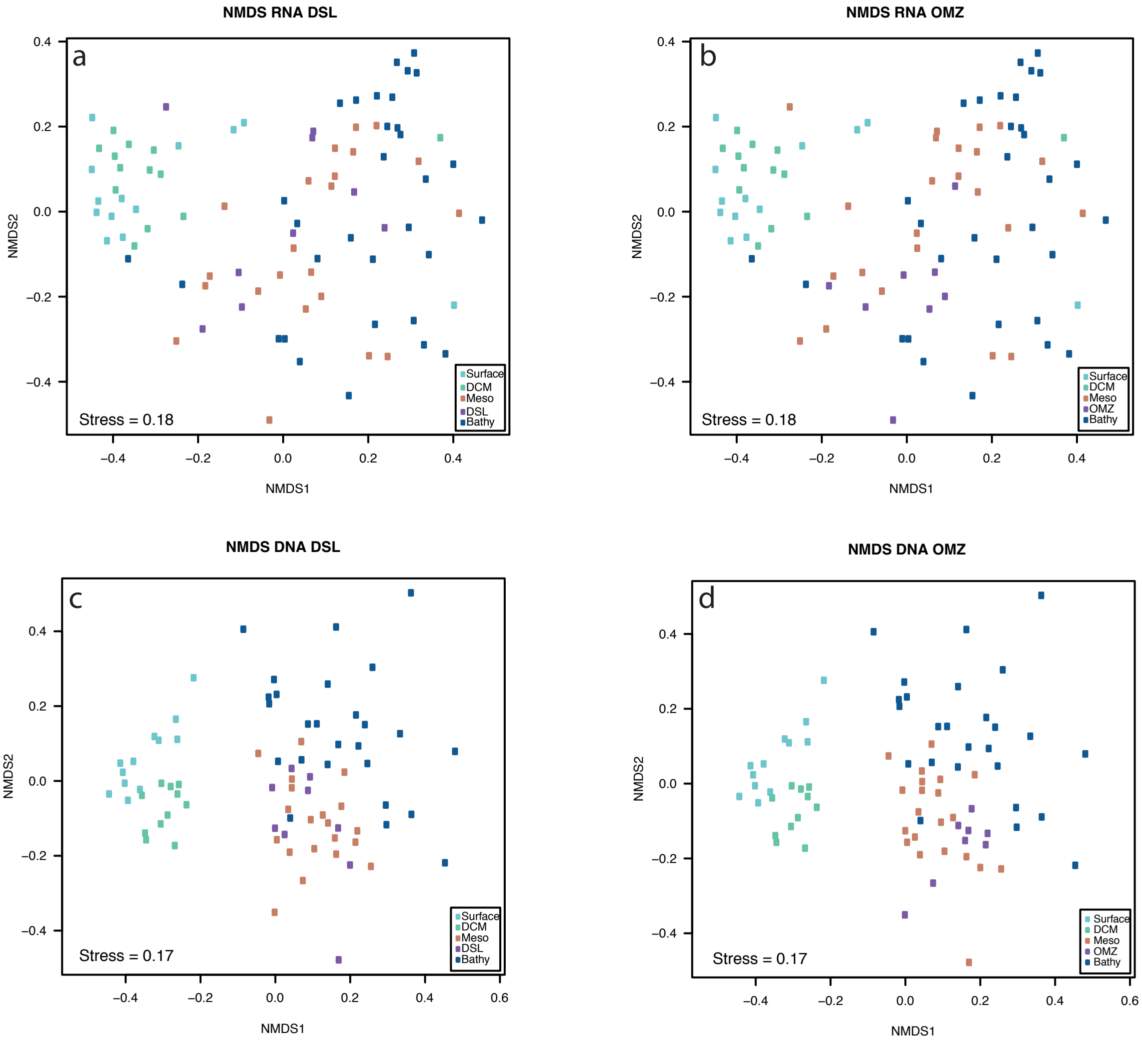

Figure S14. Richness in the different water layers defined as DSL (a) and OMZ (b). rRNA (upper boxplots) and rDNA (lower boxplots).

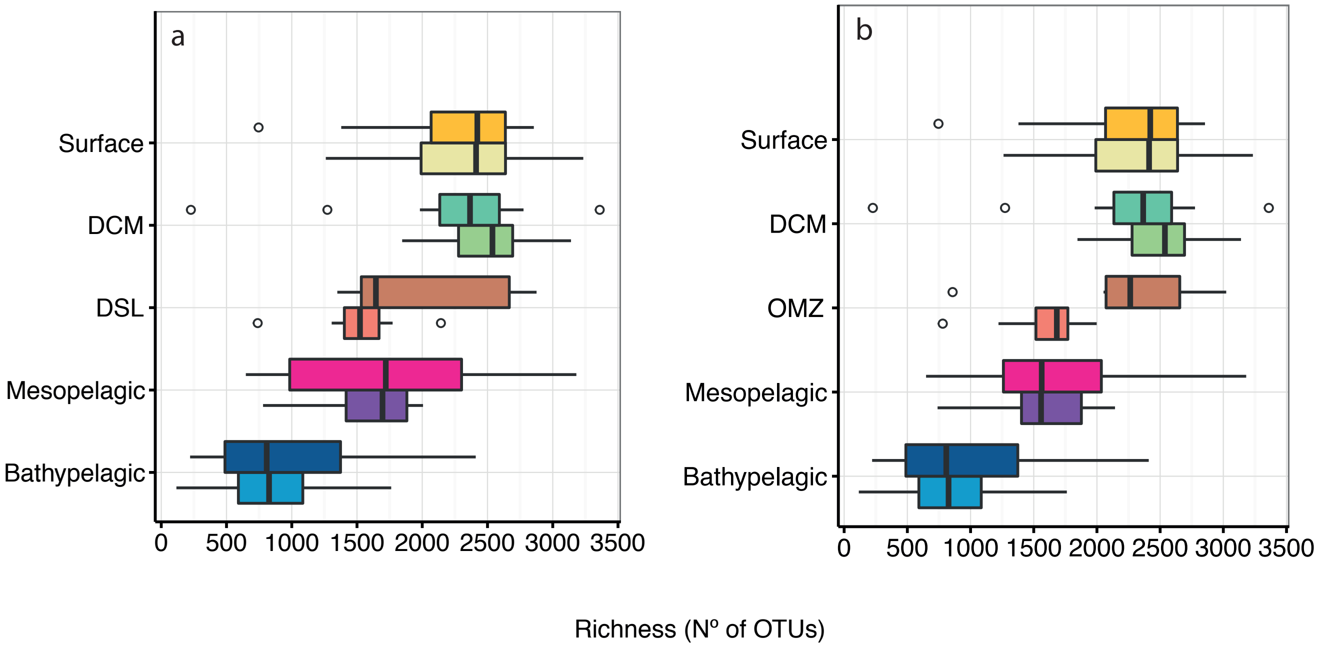

Table S1. Summary of the samples and environmental variables related.

Sample	Station	Sampling Date	Depth (m)	Latitude	Longitude	Temp. (ºC)	Salinity	O2 (ml/L)	Ocean basin	Water Mass
66	6	12/22/2010	3	23.274	-22.222	23.4	37.1	4.6	Atlantic	-
68	6	12/22/2010	4000	23.274	-22.222	2.4	34.9	5.4	Atlantic	NADW2
72	6	12/22/2010	1250	23.274	-22.222	6.6	35.3	4.0	Atlantic	NADW4.6
74	6	12/22/2010	970	23.274	-22.222	7.4	35.2	3.2	Atlantic	NADW4.6
78	6	12/22/2010	430	23.274	-22.222	13.1	35.8	3.5	Atlantic	ENACWp
80	6	12/22/2010	88	23.274	-22.222	21.5	36.9	4.6	Atlantic	-
271	18	1/3/2011	3	-4.779	-28.169	27.3	36.3	4.3	Atlantic	-
273	18	1/3/2011	4000	-4.779	-28.169	2.0	34.9	5.5	Atlantic	NADW2
275	18	1/3/2011	1700	-4.779	-28.169	4.0	35.0	5.2	Atlantic	NADW4.6
277	18	1/3/2011	800	-4.779	-28.169	4.9	34.5	3.2	Atlantic	-
279	18	1/3/2011	450	-4.779	-28.169	7.7	34.7	2.1	Atlantic	AAIW5
281	18	1/3/2011	80	-4.779	-28.169	26.3	36.3	4.4	Atlantic	-
283	18	1/3/2011	110	-4.779	-28.169	21.2	36.4	3.3	Atlantic	-
514	30	1/22/2011	3	-25.868	-27.567	25.2	36.5	4.5	Atlantic	-
516	30	1/22/2011	4000	-25.868	-27.567	1.6	34.8	5.1	Atlantic	NADW2
518	30	1/22/2011	3300	-25.868	-27.567	2.5	34.9	5.4	Atlantic	NADW2
520	30	1/22/2011	2400	-25.868	-27.567	3.0	34.9	5.3	Atlantic	NADW2
522	30	1/22/2011	850	-25.868	-27.567	4.5	34.4	4.4	Atlantic	AAIW5
524	30	1/22/2011	550	-25.868	-27.567	9.3	34.7	4.3	Atlantic	13EqAtl
526	30	1/22/2011	130	-25.868	-27.567	19.3	36.2	4.8	Atlantic	-
670	39	1/31/2011	3	-30.882	0.968	21.7	36.0	4.8	Atlantic	-
672	39	1/31/2011	4000	-30.882	0.968	1.7	34.8	4.8	Atlantic	NADW2
674	39	1/31/2011	3080	-30.882	0.968	2.3	34.9	5.0	Atlantic	NADW2
676	39	1/31/2011	1290	-30.882	0.968	3.1	34.6	3.9	Atlantic	AAIW3.1
678	39	1/31/2011	800	-30.882	0.968	4.7	34.3	4.6	Atlantic	AAIW5
680	39	1/31/2011	540	-30.882	0.968	8.5	34.6	4.0	Atlantic	AAIW5
682	39	1/31/2011	110	-30.882	0.968	16.5	35.6	5.0	Atlantic	-
862	49	2/17/2011	3	-33.907	37.042	23.5	35.7	4.6	Indian	-
864	49	2/17/2011	4000	-33.907	37.042	1.1	34.7	4.7	Indian	WSDW
866	49	2/17/2011	3000	-33.907	37.042	2.3	34.8	-	Indian	NADW2
868	49	2/17/2011	1200	-33.907	37.042	5.3	34.4	4.2	Indian	SAMW
870	49	2/17/2011	800	-33.907	37.042	10.1	34.8	4.7	Indian	SAMW
872	49	2/17/2011	450	-33.907	37.042	13.5	35.3	4.6	Indian	ICW13
874	49	2/17/2011	86	-33.907	37.042	19.2	35.6	4.6	Indian	-
999	55	2/27/2011	3	-29.354	69.400	25.3	35.6	4.4	Indian	-
1001	55	2/27/2011	4000	-29.354	69.400	1.1	34.7	4.2	Indian	CDW
1003	55	2/27/2011	2500	-29.354	69.400	1.9	34.7	3.7	Indian	CDW
1005	55	2/27/2011	1065	-29.354	69.400	4.9	34.4	4.1	Indian	AAIW3.1
1007	55	2/27/2011	715	-29.354	69.400	9.7	34.8	4.8	Indian	SAMW
1009	55	2/27/2011	400	-29.354	69.400	12.4	35.2	5.0	Indian	ICW13
1011	55	2/27/2011	130	-29.354	69.400	17.3	35.7	4.7	Indian	-
1156	63	3/7/2011	2600	-29.574	96.407	1.8	34.7	3.8	Indian	CDW
1158	63	3/7/2011	950	-29.574	96.407	5.0	34.4	4.2	Indian	AAIW3.1
1160	63	3/7/2011	1790	-29.574	96.407	2.7	34.7	3.3	Indian	CDW
1168	63	3/7/2011	650	-29.574	96.407	9.2	34.7	5.0	Indian	SAMW
1170	63	3/7/2011	3	-29.574	96.407	21.7	35.8	4.6	Indian	-
1172	63	3/7/2011	115	-29.574	96.407	16.0	35.7	5.1	Indian	-
1180	63	3/7/2011	420	-29.574	96.407	10.8	34.9	5.2	Indian	SAMW
1660	92	4/29/2011	4000	-3.410	-169.463	1.4	34.7	3.6	Pacific	CDW
1662	92	4/29/2011	1500	-3.410	-169.463	3.0	34.6	2.3	Pacific	CDW
1664	92	4/29/2011	650	-3.410	-169.463	6.7	34.6	1.7	Pacific	CDW
1666	92	4/29/2011	580	-3.410	-169.463	7.4	34.6	1.5	Pacific	13EqPac
1668	92	4/29/2011	450	-3.410	-169.463	8.5	34.7	1.7	Pacific	-
1672	92	4/29/2011	3	-3.410	-169.463	28.1	35.4	4.2	Pacific	-
1684	92	4/29/2011	65	-3.410	-169.463	28.0	35.4	4.2	Pacific	-
1758	96	5/3/2011	3	6.992	-164.372	27.4	34.8	4.3	Pacific	-
1760	96	5/3/2011	4000	6.992	-164.372	1.4	34.7	3.5	Pacific	CDW
1762	96	5/3/2011	2000	6.992	-164.372	2.2	34.7	2.2	Pacific	CDW
1764	96	5/3/2011	750	6.992	-164.372	6.0	34.6	0.8	Pacific	CDW
1766	96	5/3/2011	600	6.992	-164.372	7.0	34.6	0.6	Pacific	13EqPac
1768	96	5/3/2011	400	6.992	-164.372	8.9	34.6	0.5	Pacific	13EqPac
1770	96	5/3/2011	80	6.992	-164.372	26.4	34.8	4.2	Pacific	-
2021	110	5/23/2011	4000	17.370	-130.611	1.5	34.7	3.2	Pacific	CDW
2023	110	5/23/2011	2000	17.370	-130.611	2.1	34.6	1.9	Pacific	CDW
2025	110	5/23/2011	500	17.370	-130.611	7.3	34.5	0.2	Pacific	NPIW
2027	110	5/23/2011	400	17.370	-130.611	8.2	34.4	0.4	Pacific	NPIW
2029	110	5/23/2011	200	17.370	-130.611	11.5	34.1	3.8	Pacific	13EqPac
2031	110	5/23/2011	110	17.370	-130.611	20.1	34.7	4.6	Pacific	-
2033	110	5/23/2011	3	17.370	-130.611	21.7	34.9	4.7	Pacific	-
2217	120	6/2/2011	2800	10.759	-102.442	1.9	34.7	2.3	Pacific	CDW

2219	120	6/2/2011	2000	10.759	-102.442	2.3	34.7	1.9	Pacific	CDW
2221	120	6/2/2011	780	10.759	-102.442	5.7	34.6	0.1	Pacific	CDW
2223	120	6/2/2011	600	10.759	-102.442	7.1	34.6	0.0	Pacific	13EqPac
2225	120	6/2/2011	280	10.759	-102.442	11.1	34.8	0.1	Pacific	ENPCW
2227	120	6/2/2011	37	10.759	-102.442	26.1	34.0	3.4	Pacific	-
2229	120	6/2/2011	3	10.759	-102.442	29.3	33.5	4.2	Pacific	-
2410	128	6/21/2011	3100	14.158	-71.673	4.2	35.0	4.6	Atlantic	-
2412	128	6/21/2011	2400	14.158	-71.673	4.1	35.0	4.6	Atlantic	-
2414	128	6/21/2011	2000	14.158	-71.673	4.1	35.0	4.6	Atlantic	-
2416	128	6/21/2011	800	14.158	-71.673	6.4	34.8	2.8	Atlantic	-
2418	128	6/21/2011	460	14.158	-71.673	10.3	35.1	2.6	Atlantic	-
2420	128	6/21/2011	106	14.158	-71.673	25.3	36.7	3.6	Atlantic	-
2422	128	6/21/2011	3	14.158	-71.673	28.7	35.6	4.2	Atlantic	-
2795	141	7/5/2011	4000	26.911	-32.837	2.4	34.9	5.2	Atlantic	NADW2
2797	141	7/5/2011	2500	26.911	-32.837	3.2	35.0	5.3	Atlantic	NADW2
2799	141	7/5/2011	1000	26.911	-32.837	7.6	35.3	3.6	Atlantic	NADW4.6
2801	141	7/5/2011	900	26.911	-32.837	8.4	35.3	3.4	Atlantic	ENACWp
2803	141	7/5/2011	430	26.911	-32.837	14.5	36.0	4.0	Atlantic	ENACWt
2805	141	7/5/2011	150	26.911	-32.837	20.1	37.0	4.6	Atlantic	-
2807	141	7/5/2011	3	26.911	-32.837	23.9	37.5	4.5	Atlantic	-

Table S2. Results of the PERMANOVA analysis for the rDNA and rRNA datasets. For each dataset whole water column, and only epipelagic and deep-ocean depths had been analyzed.

	**DNA**					
	**Whole Water column**		**Surface + DCM (Epipelagic)**		**Meso + bathypelagic (Deep – Ocean)**	
	**R2**	**p-value**	**R2**	**p-value**	**R2**	**p-value**
**Light (presence/absence)**	0.14	0.001	-	-	-	-
**Temperature**	0.04	0.001	0.11	0.002	0.08	0.001
**Salinity**	0.01	0.139	0.06	0.082	0.03	0.021
**Water Mass**	-	-	-	-	**0.24**	0.045
**O_2**	0.04	0.001	0.07	0.014	0.06	0.001
**Ocean**	0.05	0.001	0.12	0.039	0.08	0.001
**Depth**	0.07	0.001	0.09	0.001	0.01	0.783
**NO_3^-**	0.01	0.511	0.04	0.374	0.02	0.275
**PO_4^{3-}**	0.02	0.042	0.04	0.740	0.02	0.105
**SiO_4**	0.01	0.445	0.04	0.704	0.02	0.386
**DAPI**	0.01	0.122	0.05	0.268	0.01	0.864
**Conductivity**	0.02	0.017	0.04	0.495	0.03	0.017

	**RNA**					
	**Whole Water column**		**Surface + DCM (Epipelagic)**		**Meso + bathypelagic (Deep – Ocean)**	
	**R2**	**p-value**	**R2**	**p-value**	**R2**	**p-value**
**Light (presence/absence)**	0.15	0.001	-	-	-	-
**Temperature**	0.03	0.001	0.08	0.045	0.05	0.001
**Salinity**	0.02	0.017	0.09	0.032	0.04	0.023
**Water Mass**	-	-	-	-	**0.25**	0.040
**O_2**	0.06	0.001	0.04	0.296	0.02	0.173
**Ocean**	0.06	0.001	0.13	0.052	0.07	0.006
**Depth**	0.07	0.001	0.11	0.004		
**NO_3^-**	0.01	0.227	0.03	0.777	0.01	0.989
**PO_4^{3-}**	0.02	0.077	0.03	0.563	0.02	0.260
**SiO_4**	0.01	0.819	0.02	0.872	0.01	0.506
**DAPI**	0.01	0.268	0.08	0.038	0.02	0.317
**Conductivity**	0.01	0.343	0.04	0.438	0.02	0.320

Table S3. Number of total OTUs in each water layer and of the unique OTUs within them in the DNA and RNA dataset.

DNA

Depth	Total OTUs	Nº of reads	OTUs unique	Nº reads unique	%reads unique
Surface	5968	259,490	765	11,221	4.3
DCM	6741	239,147	849	16,989	7.1
Mesopelagic	8106	643,049	1652	47,084	7.3
Bathypelagic	6117	578,182	584	14,549	2.5

RNA

Depth	Total OTUs	Nº of reads	OTUs unique	Nº reads unique	%reads unique
Surface	6171	285,018	386	5,415	1.9
DCM	6891	285,525	284	3,339	1.2
Mesopelagic	9622	685,314	1221	22,967	3.35
Bathypelagic	8046	711,808	314	4,510	0.6
